# Cr^3+^-Containing Carbonates and Cr_2_O_3_-*Pbcn* at Extreme Conditions

**DOI:** 10.1021/acs.inorgchem.4c05003

**Published:** 2025-03-04

**Authors:** Yu Wang, Lkhamsuren Bayarjargal, Maxim Bykov, Elena Bykova, Dominik Spahr, Konstantin Glazyrin, Victor Milman, Bjoern Winkler

**Affiliations:** † Institute of Geosciences, Goethe University Frankfurt, 60438 Frankfurt, Germany; ‡ Institute of Inorganic and Analytical Chemistry, Goethe University Frankfurt, 60438 Frankfurt, Germany; § Deutsches Elektronen-Synchrotron DESY, Notkestr. 85, 22607 Hamburg, Germany; ∥ Dassault Systemes BIOVIA, 22 Cambridge Science Park, Cambridge CB4 0FJ, U.K.

## Abstract

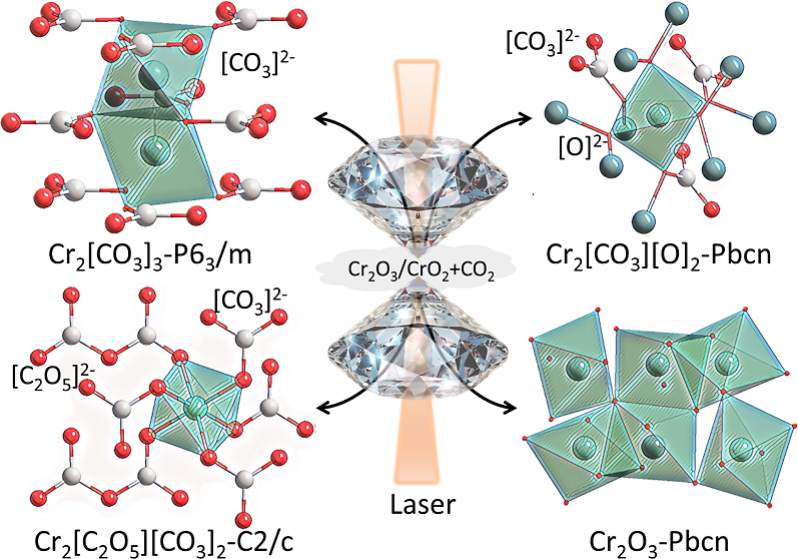

We synthesized three carbonates, Cr_2_[CO_3_]_3_, Cr_2_[C_2_O_5_][CO_3_]_2_, and Cr_2_[CO_3_][O]_2_, containing Cr^3+^ by laser heating of chromium oxides in CO_2_ at pressures between 45 and 55 GPa. All phases were characterized by single-crystal X-ray diffraction and Raman spectroscopy, while density functional theory calculations complemented the experimental results. The structure of Cr_2_[CO_3_]_3_-*P*6_3_/*m* contains coplanar groups, and a 4*f* Wyckoff site is half occupied by Cr^3+^. Cr_2_[C_2_O_5_][CO_3_]_2_-*C*2/*c* is characterized by coplanar  and pyro- anions and is isostructural to Al_2_[C_2_O_5_][CO_3_]_2_. It was obtained only after prolonged laser heating. The oxycarbonate Cr_2_[CO_3_][O]_2_-*Pbcn* was synthesized at the highest pressure (55 GPa) studied here. In this structure, half of the oxygen atoms coordinate one carbon and two Cr atoms, while the other half coordinate three chromium atoms. The oxidation state of chromium in the carbonates is +3, independent of the oxidation state (+3 or +4) of chromium in the oxide starting material. We observed the transformation of Cr_2_O_3_-*R*3̅*c* to Cr_2_O_3_-*Pbcn* above 45(2) GPa after laser heating.

## Abstract 2

We synthesized three carbonates, Cr_2_[CO_3_]_3_, Cr_2_[C_2_O_5_][CO_3_]_2_, and Cr_2_[CO_3_][O]_2_, containing Cr^3+^ by laser heating of chromium oxides in CO_2_ at pressures between 45 and 55 GPa. All phases were characterized by single-crystal X-ray diffraction and Raman spectroscopy, while density functional theory calculations complemented the experimental results.

## Introduction

The synthesis of anhydrous carbonates by reactions of oxides with CO_2_ has been pioneered about 50 years ago and led to the successful synthesis of numerous divalent 3*d*-transition metal carbonates, such as MnCO_3_, FeCO_3_, CoCO_3_, NiCO_3_, and CuCO_3_.^1,2^ However, at that time, it was not possible to carry out in situ diffraction studies at high pressures, and hence, only carbonates which could be quenched to ambient conditions could be studied.

More recently, studies examining the products of reactions between oxides and carbonates with CO_2_ induced by laser heating at high pressures after temperature quenching have revealed numerous new compounds including the first inorganic pyrocarbonates.^3−6^ We have shown that it is possible to obtain anhydrous carbonates with trivalent cations at moderate pressures and have determined the structure of Al_2_[CO_3_]_3_-*Fdd*2^7^ and Fe_2_[CO_3_]_3_-*P*2_1_/*n*.^8^ The only other chemically simple carbonates, i.e., carbonates with one type of cation, with trivalent cations are the sp^3^-hybridized carbonates Fe_4_^3+^C_3_O_12_ and Fe_2_^2+^Fe_2_^3+^C_4_O_13_, which, however, can only be obtained by laser heating at high pressures above 70 GPa.^9,10^ Furthermore, we have also obtained mixed pyrocarbonate such as Al_2_[C_2_O_5_][CO_3_]_2_-*C*2/*c* by reacting oxides with CO_2_.^7^

It is now an open question whether further chemically simple anhydrous carbonates with trivalent cations can be obtained. Here, we demonstrate that this is indeed the case by the synthesis of Cr^3+^-containing carbonates. Nanosized trivalent chromium carbonate was claimed to have been prepared by precipitation, but the structural characterization of the product phase was rather inadequate.^11^

Following the established workflow for the synthesis of new carbonates by reacting oxides with CO_2_ in a laser-heated diamond anvil cell (DAC), LH-DAC, two chromium oxides Cr^4+^O_2_, and Cr_2_^3+^O_3_ with different valences^12,13^ were employed in the synthesis, thus offering the opportunity to also study the influence of the valence state of the starting material.

## Method

### High-Pressure Synthesis Experiments

Boehler–Almax design^14^ DACs equipped with 300 μm culets were used to generate high pressures. Rhenium gaskets were preindented to a thickness of 40–50 μm and laser drilled to form sample chambers with diameters of 100–120 μm. CrO_2_ (99.9%) powder purchased from Aladdin (CAS No. 12018-01-8) and Cr_2_O_3_ (99.9%) powder purchased from Sigma-Aldrich (CAS No. 1308-38-9) were placed into the sample chambers of different DACs, respectively. CO_2_ dry ice was then cryogenically loaded into the gasket hole under an inert argon atmosphere to minimize contamination with H_2_O during the process.^3,15^ In our experiments, CO_2_ served not only as a pressure-transmitting medium but also as a chemical reactant with chromium oxides at high pressures and high temperatures. The edge of the diamond Raman band measured at the center of diamond culet was used to estimate the pressure before laser heating.^16^ After stepwise compression to targeted pressures, the samples were heated from both sides using a near-infrared (NIR) fiber laser (λ = 1064 nm) at DESY or CO_2_ laser (coherent Diamond K-250, λ = 10.6 μm) in our laboratory in Frankfurt (Germany).^17^ After a brief laser heating, the sample was quenched to an ambient temperature. The pressure was remeasured after laser heating by determining the characteristic frequency of the ν_1_-vibration of CO_2_–V.^18^ The accuracy of the pressure determination given in this work is estimated to be ≈ 7%. Raman spectra and X-ray diffraction data were collected before and after laser heating.

### High-Pressure Raman Experiments

Raman spectra were collected after laser heating in the DAC with an Alpha300 R—Raman Imaging Microscope from Oxford Instruments WITec.^19^ This system was equipped with a 532 nm excitation laser, a thermoelectrically cooled CCD detector, an Olympus SLMPL 50× objective, and 600/1800/2400 grooves/mm BLZ gratings. Here, we mainly choose the 600/1800 grooves/mm gratings to acquire the Raman frames from a large area by measuring spectra on a 100 × 100 μm^2^ with 2 μm step.

### High-Pressure XRD Experiments

X-ray diffraction experiments were performed at the extreme conditions beamline P02.2 at PETRA III (DESY, Hamburg, Germany).^20−22^ Radiation with a wavelength of 0.29 Å and a beam size of ≈2 × 2 μ m was used for X-ray diffraction measurements. The mixture of chromium oxides and CO_2_ at high pressures before laser heating was characterized by powder X-ray diffraction measurements performed by using a PerkinElmer XRD 1621 detector. The sample-to-detector geometry was calibrated with a CeO_2_ standard powder, and the powder pattern was integrated using Dioptas.^23^ The powder XRD pattern was indexed and analyzed employing GSASII.^24^

At high pressure, microscale-sized single crystals were grown, and SCXRD measurements on the laser-heated sample were collected. The instrumental parameters were calibrated with an *ortho*-enstatite crystal ((Mg_1.93_, Fe_0.06_)(Si_1.93_, Al_0.06_)O_6_-*Pbca*, *a* = 8.8117(2) Å, *b* = 5.18320(10) Å, and *c* = 18.2391(3) Å). The laser-heated DACs were rotated up to ±30° around the axis perpendicular to the beam, and all frames were collected in 0.5° step scans with 5s acquisition time per frame. For the analysis of the single-crystal diffraction data (indexing, data integration, frame scaling, and absorption correction), we used the CrysAlisPro software package in conjunction with the DAFi package.^25^ Absorption correction was performed using spherical harmonics implemented in the SCALE3 ABSPACK scaling algorithm. Using the Olex2 crystallography software package,^26^ the structures were solved with the ShelXT structure solution program^27^ and refined with the ShelXL.^28^

### Density Functional Theory-Based Calculations

First-principles calculations were carried out within the framework of density functional theory (DFT), employing the Perdew–Burke–Ernzerhof (PBE) exchange–correlation functional and the plane wave/pseudopotential approach implemented in the CASTEP simulation package.^29−31^ “On the fly” norm-conserving or ultrasoft pseudopotentials generated using the descriptors in the CASTEP database were employed in conjunction with plane waves up to a kinetic energy cutoff of 1020 or 630 eV, for norm-conserving and ultrasoft pseudopotentials, respectively. The accuracy of the pseudopotentials is well established.^32^ A Monkhorst–Pack grid was used for Brillouin zone integrations.^33^ We used a distance between grid points of <0.023 2π/Å. Convergence criteria for geometry optimization included an energy change of <5 × 10^–6^ eV atom^–1^ between steps, a maximal force of <0.008 eV Å^–1^, and a maximal component of the stress tensor of <0.02 GPa. Spin-polarized calculations were carried out both with and without a local Coulomb correction (+*U*). The CASTEP implementation of DFT + *U* is based on a simplified, rotationally invariant approach.^34,35^ The only external parameter required for this approach is the effective value of the on-site Coulomb parameter, *U*, for each affected orbital. Here, the effective value for the d-orbitals of Cr was set to 2.5 eV. Phonon frequencies were obtained from density functional perturbation theory (DFPT) calculations.^36,37^ Raman intensities were computed using DFPT with the “2*n* + 1” theorem approach.^38^

## Results

### Laser Heating of CrO_2_ in CO_2_

**Figure 1:**
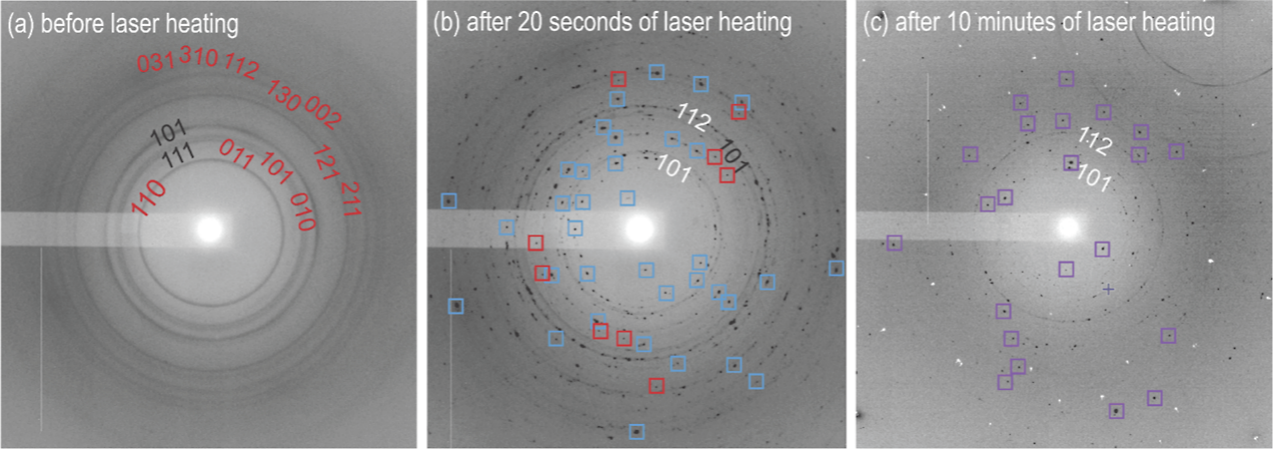
Selected 2D diffraction images of (a) CrO_2_ and CO_2_ before laser heating, (b) after 20 s of laser heating, and (c) after 10 min of laser heating at 45(2) GPa. (a) Powder rings due to CrO_2_ and CO_2_–III are labeled with Laue indices in red and black, respectively, and were collected before laser heating. (b) Appearance of diffraction spots marked by blue squares indicates the occurrence of a reaction. Red squares mark diffraction spots of CrO_2_. The hkl indices belonging to CO_2_–III are shown in black, while those assigned to CO_2_–V are shown in white. (c) Purple squares indicate diffraction spots collected on Cr_2_[C_2_O_5_][CO_3_]-*C*2/*c*, and numbers designate hkl-indices of CO_2_–V (white).

Before laser heating, the 1D integrated pattern could be indexed with the structure models (Figure S1) and 2D image (Figure 1a) of CrO_2_-*Pnnm*^12^ and CO_2_-*Cmca* (CO_2_–III).^39^ The lattice parameters of CrO_2_ at 45(2) GPa are *a* = 4.035(3) Å, *b* = 4.473(9) Å, and *c* = 2.782(4) Å. At the same pressure for CO_2_–III, we obtained *a* = 4.029(1) Å, *b* = 4.144(3) Å, and *c* = 5.854(3) Å. These values are consistent with those reported previously.^12,40^

During laser heating of CrO_2_ in CO_2_ to a temperature of about 2000 K at 45(2) GPa, the opaque CrO_2_ powder started to couple with the laser and react with CO_2_ surrounding it. The formation of two new phases led to new reflections, marked by blue and purple squares in Figure 1b,c. CO_2_–III transformed into CO_2_–V.^39,41^

**Figure 2:**
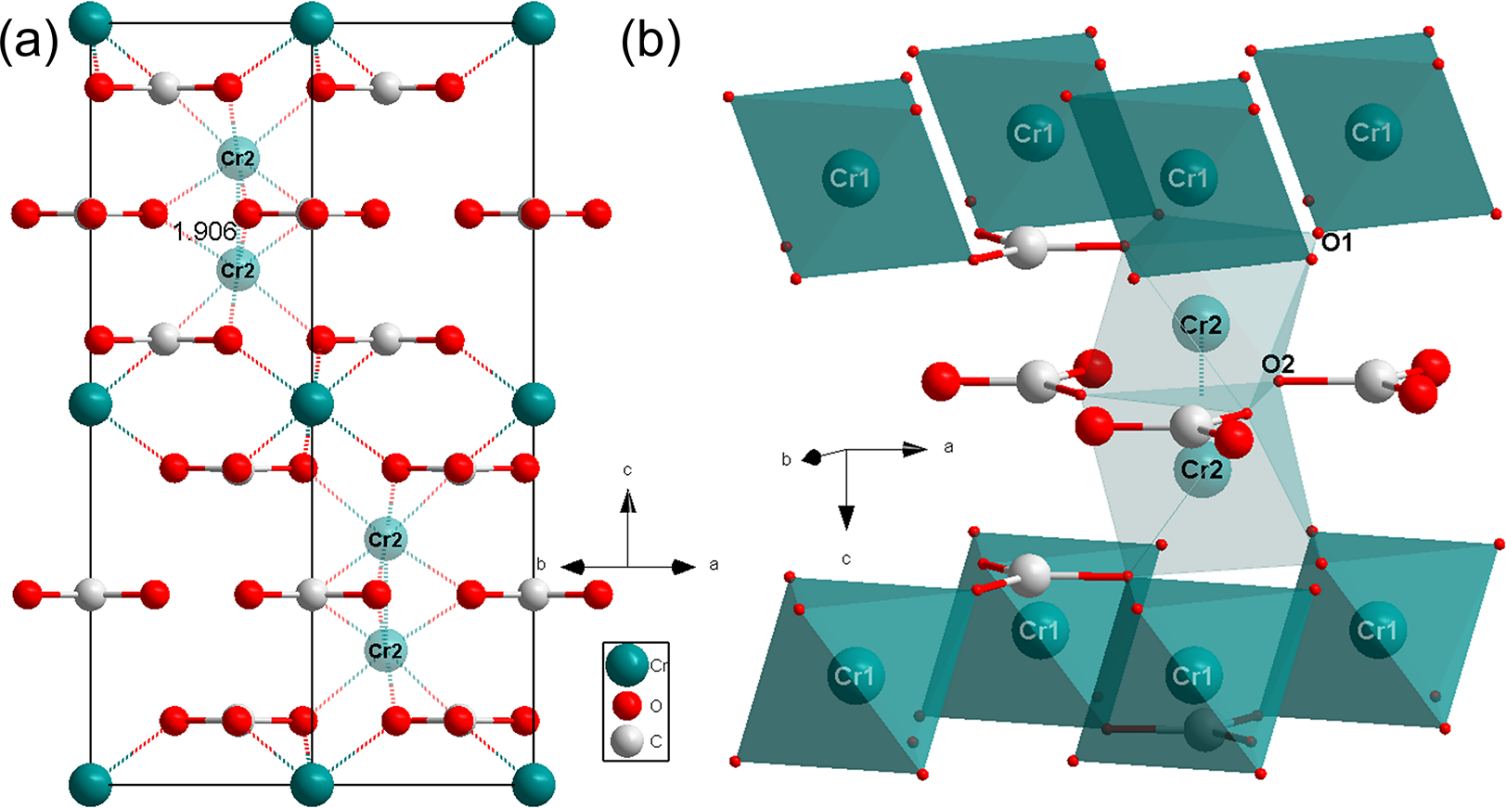
(a) Crystal structures of Cr_2_[CO_3_]_3_-*P*6_3_/*m* at 45(2) GPa viewed along the [110] direction, showing the planar arrangement of the  groups. (b) Octahedra and their neighbored  groups. The Cr2 positions are only half-occupied.

The analysis of the single-crystal X-ray diffraction data showed that one of the new phases in Figure 1b is Cr_2_[CO_3_]_3_-*P*6_3_/*m*, which has *Z* = 2 formula units in the unit cell (Figures 2a and S2 and Table S1). The lattice parameters of Cr_2_[CO_3_]_3_-*P*6_3_/*m* at 45(2) GPa are *a* = *b* = 4.375(4) Å and *c* = 13.021(10) Å. Cr_2_[CO_3_]_3_ contains parallel  layers. The C–O bond lengths are 1.25(7) and 1.26(10) Å. Cr atoms occupying the 2*b* Wyckoff position (Table S2), labeled as Cr1, are coordinated by six oxygen atoms at a distance of 1.91(9) Å (Figure 2b) and form an undistorted octahedron. The Cr atoms on the 4*f* Wyckoff position are labeled as Cr2. The distances between Cr2–O1 and Cr2–O2 are 1.96(3) and 1.85(5) Å, resulting in a distorted octahedron (Figure 2b). The octahedron around a Cr2 atom shares a face with its neighboring Cr2 octahedron (Figure 2b). The distance between the two nearest Cr2 atoms is 1.90(6) Å (Figure 2a). Although very short Cr–Cr bonds of 1.8028(9) Å have been reported,^42^ an alternative is a structural model where the Cr2 sites are partially occupied with a site occupied factor of 0.5. Such a model results in a significant improvement in the *R*_1_ value during the refinement from 14.3% down to 6.35%. The chemical composition of this positionally disordered phase is Cr_2_[CO_3_]_3_, consistent with Cr^3+^ carbonate.

**Figure 3:**
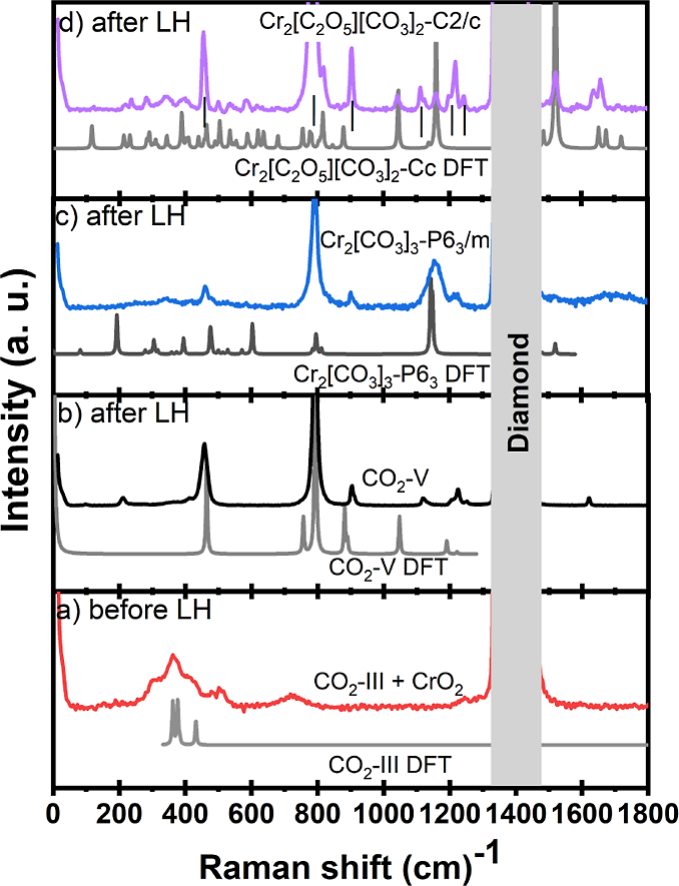
Raman spectra measured before (a) and after (b–d) laser heating at 45(2) GPa at different locations in the sample chamber show the simultaneous presence of CO_2_–V, Cr_2_[CO_3_]_3_-*P*6_3_/*m* and Cr_2_[C_2_O_5_][CO_3_]_2_-*C*2/*c*. (a) Experimental Raman spectrum of CrO_2_ and calculated Raman of CO_2_–III. (b) Raman spectra of CO_2_–V by experiments (black) and theory (gray). (c) Raman spectra of Cr_2_[CO_3_]_3_-*P*6_3_/*m* by experiments (blue) and Cr_2_[CO_3_]_3_-*P*6_3_ by DFT (gray). (d) Raman spectra of Cr_2_[C_2_O_5_][CO_3_]_2_-*C*2/*c* by experiments (purple) and Cr_2_[C_2_O_5_][CO_3_]_2_-*Cc* by DFT (gray).

The Raman spectra of CrO_2_ and CO_2_ before and after laser heating are presented in Figure 3. We could observe only a broad C–O stretching band at around 1150 cm^–1^ from the Raman spectrum of Cr_2_[CO_3_]_3_-*P*6_3_/*m* (Figure 3c).

**Figure 4:**
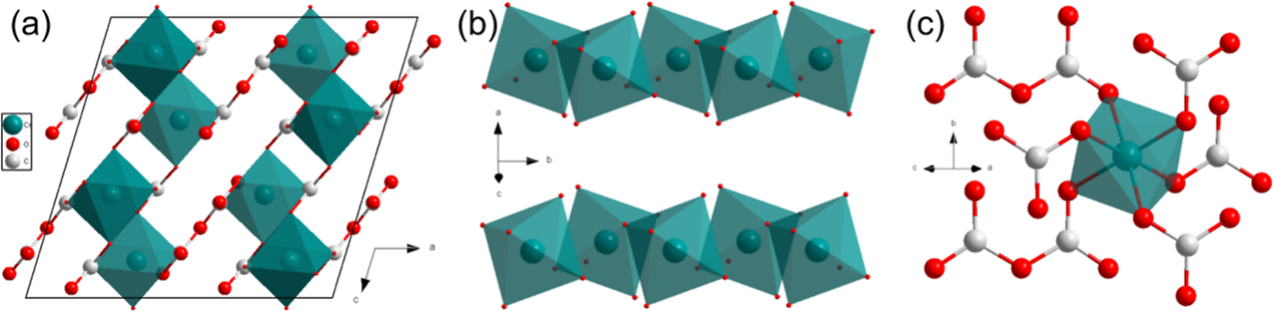
(a) Crystal structure of Cr_2_[C_2_O_5_][CO_3_]_2_-*C*2/*c* at 45(2) GPa view along the *b* axis, displaying coplanar trigonal - and pyrocarbonate - anions. (b) CrO_6_ octahedral chain by sharing corners along the *b* axis. (c) CrO_6_ connected with  and  groups viewed along the [101] direction.

After prolonged heating (10 min) on CrO_2_*at* a different sample position in CO_2_ at 45(2) GPa, the second phase Cr_2_[C_2_O_5_][CO_3_]_2_ was synthesized. It was identified by the appearance of additional bands in the Raman spectrum in the frequency ranges of 200–600, 800–1200, and 1500–1700 cm^–1^, as shown in Figure 3d. Single-crystal X-ray diffraction data were collected on a temperature-quenched sample (Figure 1c), and the chemical formula was derived from the structure solution (Figure 4 and Table S3). Cr_2_[C_2_O_5_][CO_3_]_2_-*C*2/*c* contains both conventional trigonal - and pyrocarbonate - anions and is isostructural to the recently reported Al_2_[C_2_O_5_][CO_3_]_2_.^7^ At 45(2) GPa, the lattice parameters of Cr_2_[C_2_O_5_][CO_3_]_2_-*C*2/*c* are *a* = 11.381(4), *b* = 4.296(10), and *c* = 10.870(9) Å, β = 106.9(9)°, respectively (Table S3). The - and pyrocarbonate -groups are aligned forming parallel planes (Figure 4a,c). The CrO_6_-octahedra form chains along the *b* axis by sharing oxygen atoms (Figure 4b). The average Cr–O bond length is ∼1.89(2) Å (Figure 4d). The C–O distances in trigonal  vary from 1.24 to 1.29 Å, whereas in , they range from 1.22 to 1.34 Å.

### Laser Heating of Cr_2_O_3_ in CO_2_

To better understand the role of the Cr-oxidation state during the synthesis of chromium carbonates, we also performed experiments by laser heating Cr_2_O_3_ in CO_2_, thus changing the oxidation state of the Cr cation from +4 to +3.

**Figure 5:**
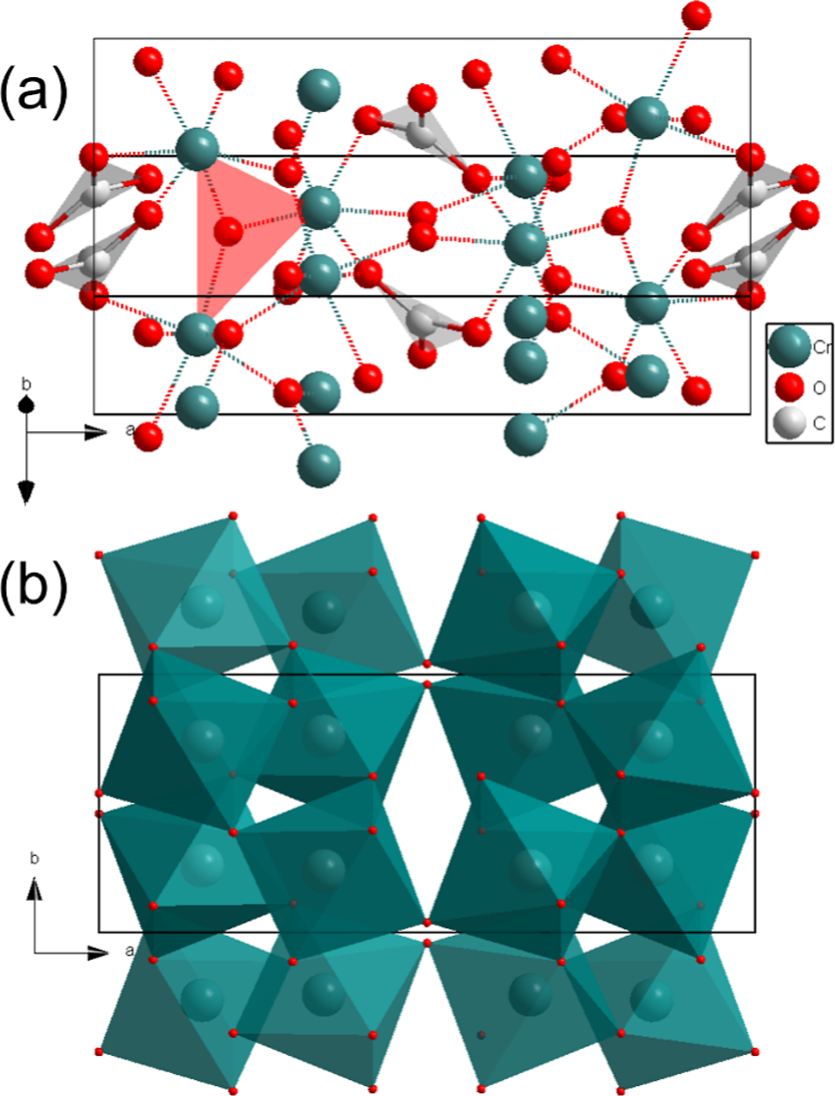
(a) Crystal structure of Cr_2_[CO_3_][O]_2_-*Pbcn* at 55(2) GPa, displaying the  groups in gray triangles and OCr_3_ triangles in red in the unit cell. (b) CrO_6_ octahedra form in chains by sharing corners along the *c* axis.

On laser-heated Cr_2_O_3_ in CO_2_ at 55(2) GPa, we observed the appearance of new sharp spots in the diffraction pattern. Single-crystal X-ray diffraction data were collected on a temperature-quenched sample. Reflections of one of the phases at 50(2) GPa could be indexed using an orthorhombic unit cell (space group *Pbcn*) with *a* = 11.195(1), *b* = 4.410(1), and *c* = 4.945(7) Å (Figure 5). A structure solution identified this phase as a further novel chromium oxycarbonate with composition Cr_2_[CO_3_][O]_2_, which also contains  layers in angle planes (Figure 5a) The C–O bonds are about 1.26–1.27 Å length. The oxygen atoms in this phase are 3-fold coordinated, half of them connected with three Cr atoms and half of them connected with two Cr atoms and one carbon atom (Figure 5a). CrO_6_-octahedra are distorted and are connected with each other by sharing corners forming infinite chains along the *c* axis (Figure 5b), and the same in the *b* axis direction. The structural details together with the DFT calculations are summarized in Table S4.

**Figure 6:**
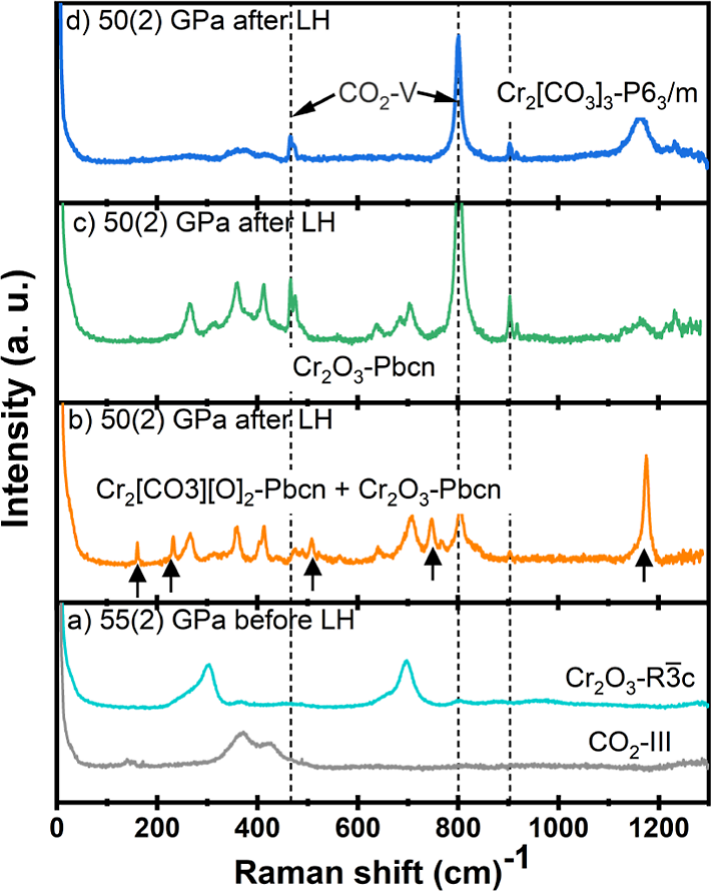
Raman spectra of Cr_2_O_3_ and CO_2_ measured before (a) and after (b–d) laser heating. The dashed lines mark the positions assigned to CO_2_–V after laser heating. (a) Raman spectra of Cr_2_O_3_-*R*3̅*c* and CO_2_–III before laser heating at 55(2) GPa. (b) Raman spectra of Cr_2_[CO_3_][O]_2_-*Pbcn* and Cr_2_O_3_-*Pbcn* after laser heating at 50(2) GPa. The arrows are marked to the Raman frequencies of Cr_2_[CO_3_][O]_2_-*Pbcn*. (c) Raman spectrum in green represents the mixture of Cr_2_O_3_-*Pbcn* and Cr_2_O_3_-*R*3̅*c* collected in the experiments. (d) Raman spectrum of Cr_2_[CO_3_]_3_-*P*6_3_/*m* phase.

The changes in the Raman spectrum of the mixture of Cr_2_O_3_ and CO_2_ due to laser heating at 55(2) GPa are presented in Figure 6. New Raman bands due to Cr_2_[CO_3_][O]_2_ appear from 160 to 1180 cm^–1^ (Figure 6b).

### DFT Calculations

The DFT + *U* full geometry optimizations gave structures in good agreement with the experimental findings. The partially disordered structure of Cr_2_[CO_3_]_3_-*P*6_3_/*m* was approximated by an ordered structure with the space group symmetry *P*6_3_. The computed lattice parameters agreed with the experimental values within 1–2.5%, which is typical for calculations such as those carried out here. The enthalpy difference between a ferromagnetic and an antiferromagnetic spin arrangement was too small (0.01 eV per unit cell) to draw a conclusion regarding the preferred spin arrangement. Similarly, for Cr_2_[C_2_O_5_][CO_3_]_2_-*C*2/*c* and Cr_2_[CO_3_][O]_2_-*Pbcn*, the computed lattice parameters also agreed with the experimental values at 45 GPa to within 1.2%.

### Cr_2_O_3_

**Figure 7:**
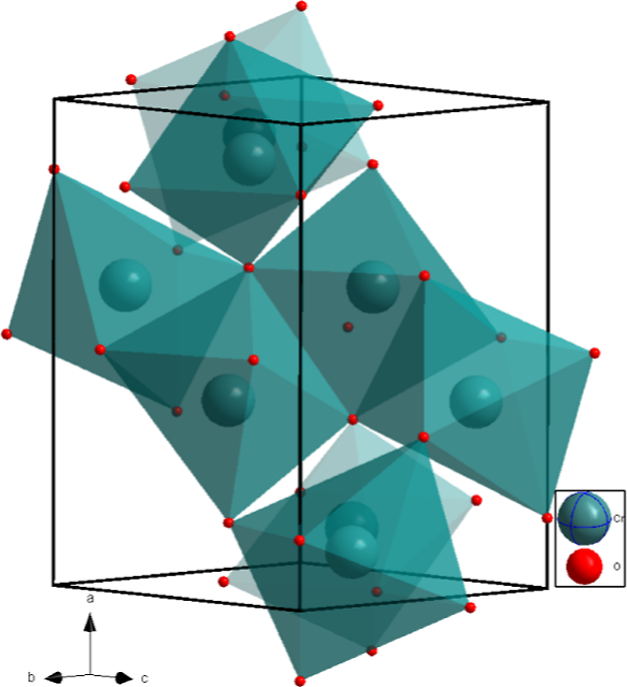
Crystal structure of Cr_2_O_3_-*Pbcn* after laser heating at 50(2) GPa, showing that the octahedra of the CrO_6_ units in a unit cell are connected by sharing corners, edges, and faces.

In the experiments where we heated Cr_2_O_3_, we observed an additional phase transition. Cr_2_O_3_-*R*3̅*c* has characteristic Raman bands at 300 cm^–1^ (*E*_g_) and 690 cm^–1^ (*A*_1g_) (Figure 6a). The appearance of new bands at 265, 360, and 412 cm^–1^ in the Raman spectra after laser heating indicated the occurrence of an additional phase (Figures 6c and S3). The high quality of the SCXRD data allowed for the unambiguous identification of this phase as Cr_2_O_3_-*Pbcn*. The structure of Cr_2_O_3_-*Pbcn* contains significantly distorted polyhedra, and the octahedra form a 3D network by sharing corners, edges, and faces (Figure 7). The refinement details at 50(2) GPa are summarized in Table S5, where they are compared to the results of the DFT calculations. The good agreement between the two data sets supports the result of the structure solution and refinement (Table S8).

### Heating on Decompression

**Figure 8:**
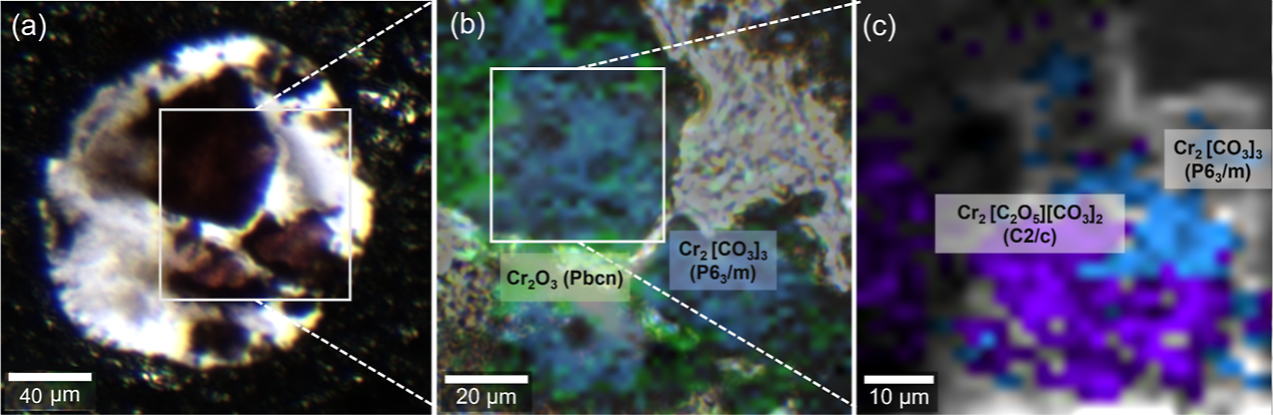
(a) Microimage of Cr_2_O_3_ and CO_2_ in a DAC. The white solid square indicates the first heated and mapping area at 45(2) GPa displayed in (b). (b) Combination of Raman and sample image layers after first laser heating (∼10*s*) at 45(2) GPa. The white square marked the second heated and mapping area displayed in (c). The phases are labeled with Cr_2_O_3_-*Pbcn* in green and Cr_2_[CO_3_]_3_-*P*6_3_/*m* in blue, which are featured by a frequency of 408 and 1150 ± 10 cm^–1^, respectively. (c) Combination of Raman and sample image layers after second laser heating (∼20 min) at 45(2) GPa. The phase Cr_2_[C_2_O_5_][CO_3_]_2_-*C*2/*c* marked in purple is featured by a frequency of 1045 ± 10 cm^–1^.

In the temperature-quenched sample at 50(2) GPa, we observed, in addition to Cr_2_[CO_3_][O]_2_-*Pbcn* and Cr_2_O_3_-*Pbcn*, also Cr_2_[CO_3_]_3_-*P*6_3_/*m* (Figure 6d), which we had synthesized earlier from CrO_2_ and CO_2_ at 45(2) GPa. We decompressed this sample from 50(2) to 45(2) GPa and laser heated again. Upon laser heating at 45(2) GPa, Cr_2_[CO_3_][O]_2_-*Pbcn* disappeared, Cr_2_[CO_3_]_3_-*P*6_3_/*m* remained in the sample, and after prolonged heating, Cr_2_[C_2_O_5_][CO_3_]_2_-*C*2/*c* formed. The changes in this sample were characterized by Raman map, as it is shown in Figure 8.

## Discussion

We have shown that at moderately high pressures, Cr^3+^-containing carbonates can be formed. These are the first single-crystal structure refinement of Cr-carbonates, whose existence further extends the chemical diversity of the family of anhydrous chemically simple carbonates. For the conditions employed here, the valence state of Cr in the starting material seems to be irrelevant (Table S7), as in all cases, the resulting carbonate contained Cr^3+^.

**Figure 9:**
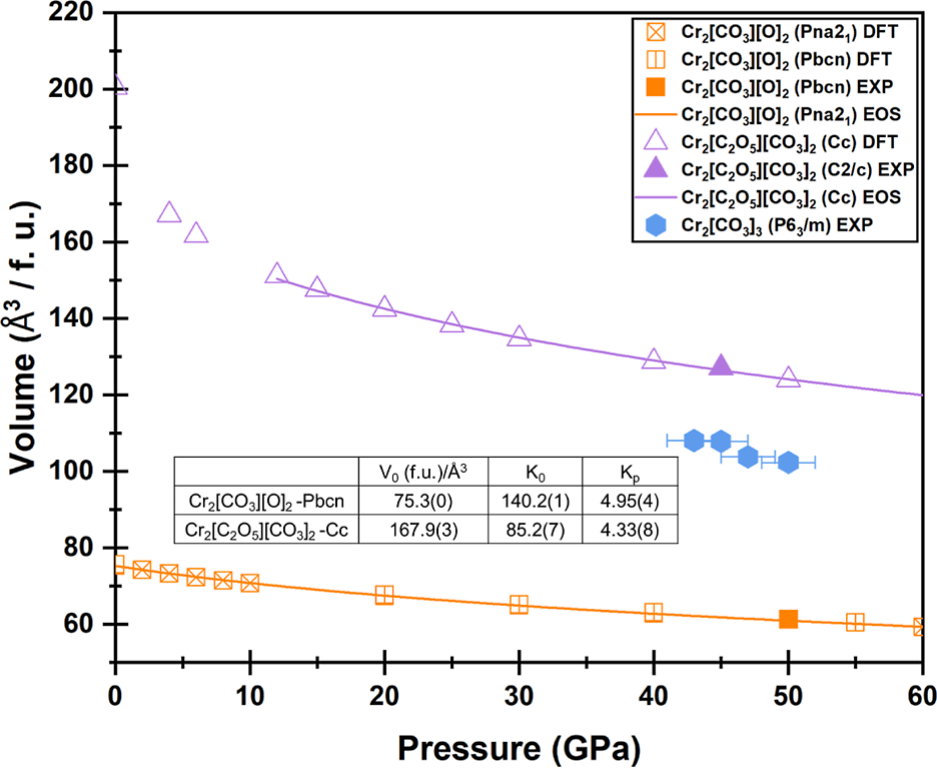
Equation of the state of the carbonates at high pressures by DFT^45^ shown together with experimental data.

The ionic radii of Fe^3+^, Cr^3+^, and Al^3+^ in 6-fold coordination are 0.645 Å, 0.615 Å, and 0.535 Å, respectively.^43^ Hence, the radii of Cr^3+^ and Al^3+^ differ by ≈ 15%, which is often taken as a limit up to which substitution and solid solution formation is facile. However, Fe_2_[CO_3_]_3_-*P*2_1_/*n*, Cr_2_[CO_3_]_3_-*P*6_3_/*m*, and Al_2_[CO_3_]_3_-*Fdd*2 all crystallize in different structure types. In contrast, Al_2_[C_2_O_5_][CO_3_]_2_ and Cr_2_[C_2_O_5_][CO_3_]_2_ are isostructural in *C*2/*c* space group symmetry. The volume of Cr_2_[C_2_O_5_][CO_3_]_2_ is 508.62(2) Å^3^ and hence larger than that of Al_2_[C_2_O_5_][CO_3_]_2_, which is 477.8 Å^3^ at almost the same pressure, consistent with the larger ionic radius of Cr^3+^ relative to Al^3+^. The geometric parameters of the two phases generated^44^ are shown in Table S6. The *p*–V equations of states of the carbonates synthesized in this work are shown in Figure 9.

There has been a long discussion about whether there is an orthorhombic phase of Cr_2_O_3_ above 30 GPa.^13,46−48^ In our DFT calculations of the enthalpy difference between Cr_2_O_3_-*Pbcn* and Cr_2_O_3_-*R*3̅*c*, the high-pressure orthorhombic polymorph becomes more stable above 15 GPa (Figure S4). This is in agreement with earlier and similar DFT-based studies.^49,50^ However, in these calculations, the temperature is neglected. Without laser heating, Cr_2_O_3_-*R*3̅*c* can be cold-compressed up to 70 GPa.^13,47^ In the current study, the formation of Cr_2_O_3_-*Pbcn* was observed at just two *p, T*-points at 45 and 50 GPa, and hence the stability field cannot be inferred, but we provide the first experimental structure of the *Pbcn* phase (Figure 7 and Figure S5).

The transition pressure from corundum Al_2_O_3_-*R*3̅*c* to Al_2_O_3_-*Pbcn* occurs at ≈80–100 GPa upon heating.^51−53^ The same transition for Fe_2_O_3_-*R*3̅*c* to Fe_2_O_3_-*Pbcn* takes place at significantly lower pressure of ≈40 GPa.^54^ As Fe^3+^ and Cr^3+^ have a similar electronic configuration and size, it is reasonable to ensure that the transition for Cr_2_O_3_-*R*3̅*c* also takes place at around 40 GPa, which is consistent with our observations (Table S9).

## Conclusions

This study demonstrates the successful high-pressure synthesis of three novel chromium(III)-containing carbonates—Cr_2_[CO_3_]_3_, Cr_2_[C_2_O_5_][CO_3_]_2_, and Cr_2_[CO_3_][O]_2_—and a high-pressure polymorph Cr_2_O_3_-*Pbcn*. Using state-of-the-art single-crystal diffraction at high pressures, we clarified that both sp^2^ and pyrocarbonates containing trivalent Cr ions can be obtained by inducing reactions in DACs by laser heating. The oxidation state of chromium in the carbonates is +3, independent of the oxidation state (+3 or +4) of chromium in the oxide starting material. We also observed the transformation of Cr_2_O_3_-*R*3̅*c* to Cr_2_O_3_-*Pbcn* above 45(2) GPa after laser heating. In the current study, we did not explore if the samples are quenchable to ambient conditions and their stability. This is planned for a future study.

## Supporting Information Available

Additional experimental and theory data, fractional atomic coordinates and isotropic displacement parameters, Raman spectra of Cr_2_O_3_ compared in experiment and DFT, equation of the state of Cr_2_O_3_ at high pressures by DFT, summary of high-pressure reactions and products, and high-pressure behavior of Cr_2_O_3_ in previous study and this work (PDF)

## References

[ref1] Seidel H.; Ehrhardt H.; Viswanathan K.; Johannes W. Darstellung, Struktur und Eigenschaften von Kupfer(II)-Carbonat. Z. fur Anorg. Allg. Chem. 1974, 410, 138–148. 10.1002/zaac.19744100207.

[ref2] Ehrhardt H.; Seidel H.; Schweer H. Hochdrucksynthesen einiger Carbonate mit überkritischem CO_2_. Z. fur Anorg. Allg. Chem. 1980, 462, 185–198. 10.1002/zaac.19804620121.

[ref3] Spahr D.; König J.; Bayarjargal L.; Luchitskaia R.; Milman V.; Perlov A.; Liermann H.-P.; Winkler B. Synthesis and structure of Pb[C_2_O_5_]: an inorganic pyrocarbonate salt. Inorg. Chem. 2022, 61, 9855–9859. 10.1021/acs.inorgchem.2c01507. 35730801

[ref4] Spahr D.; König J.; Bayarjargal L.; Milman V.; Perlov A.; Liermann H.-P.; Winkler B. Sr[C_2_O_5_] is an inorganic pyrocarbonate salt with [C_2_O_5_]^2–^ complex anions. J. Am. Chem. Soc. 2022, 144, 2899–2904. 10.1021/jacs.2c00351. 35134291

[ref5] Spahr D.; Bayarjargal L.; Haussühl E.; Luchitskaia R.; Friedrich A.; Milman V.; Fedotenko T.; Winkler B. Twisted [C_2_O_5_]^2-^-groups in Ba[C_2_O_5_] pyrocarbonate. Chem. Commun. 2023, 59, 11951–11954. 10.1039/D3CC03324D. 37747265

[ref6] Spahr D.; Bayarjargal L.; Bykov M.; Brüning L.; Reuter T. H.; Milman V.; Liermann H.-P.; Winkler B. High-pressure synthesis of acentric sodium pyrocarbonate, Na_2_[C_2_O_5_ ]. Dalton Trans. 2023, 53, 40–44. 10.1039/D3DT03673A. 38054559

[ref7] Bayarjargal L.; Spahr D.; Milman V.; Marquardt J.; Giordano N.; Winkler B. Anhydrous aluminum carbonates and isostructural compounds. Inorg. Chem. 2023, 62, 13910–13918. 10.1021/acs.inorgchem.3c01832. 37579301

[ref8] Bayarjargal L.; Spahr D.; Bykova E.; Wang Y.; Giordano N.; Milman V.; Winkler B. High-Pressure Synthesis of an Iron Carbonate, Fe_2_ [CO_3_ ]_3_. Inorg. Chem. 2024, 63, 21637–21644. 10.1021/acs.inorgchem.4c03177. 39466183

[ref9] Albers C.; et al. Fe^3+^-hosting carbon phases in the deep Earth. Phys. Rev. B 2022, 105, 085155 10.1103/PhysRevB.105.085155.

[ref10] Cerantola V.; Bykova E.; Kupenko I.; Merlini M.; Ismailova L.; McCammon C.; Bykov M.; Chumakov A. I.; Petitgirard S.; Kantor I.; et al. Stability of iron-bearing carbonates in the deep Earth’s interior. Nat. Commun. 2017, 8, 15960 10.1038/ncomms15960. 28722013 PMC5524932

[ref11] Rahimi-Nasarabadi M.; Ahmadi F.; Hamdi S.; Eslami N.; Didehban K.; Ganjali M. R. Preparation of nanosized chromium carbonate and chromium oxide green pigment through direct carbonation and precursor thermal decomposition. J. Mol. Liq. 2016, 216, 814–820. 10.1016/j.molliq.2016.01.065.

[ref12] Maddox B. R.; Yoo C. S.; Kasinathan D.; Pickett W. E.; Scalettar R. T. High-pressure structure of half-metallic CrO_2_. Phys. Rev. B 2006, 73, 144111 10.1103/PhysRevB.73.144111.

[ref13] Dera P.; Lavina B.; Meng Y.; Prakapenka V. B. Structural and electronic evolution of Cr_2_O_3_ on compression to 55 GPa. J. Solid State Chem. 2011, 184, 3040–3049. 10.1016/j.jssc.2011.09.021.

[ref14] Boehler R. New diamond cell for single-crystal X-ray diffraction. Rev. Sci. Instrum. 2006, 77, 115103 10.1063/1.2372734.

[ref15] Scelta D.; Ceppatelli M.; Ballerini R.; Hajeb A.; Peruzzini M.; Bini R. Spray-loading: a cryogenic deposition method for diamond anvil cell. Rev. Sci. Instrum. 2018, 89, 053903 10.1063/1.5011286. 29864887

[ref16] Akahama Y.; Kawamura H. Pressure calibration of diamond anvil Raman gauge to 310 GPa. J. Appl. Phys. 2006, 100, 043516 10.1063/1.2335683.

[ref17] Bayarjargal L.; Fruhner C.-J.; Schrodt N.; Winkler B. CaCO_3_ phase diagram studied with Raman spectroscopy at pressures up to 50 GPa and high temperatures and DFT modeling. Phys. Earth Planet. Inter. 2018, 281, 31–45. 10.1016/j.pepi.2018.05.002.

[ref18] Wang Y.; Bayarjargal L.; Bykova E.; Spahr D.; Bykov M.; Milman V.; Giordano N.; Fedotenko T.; Liermann H.-P.; Winkler B. Synthesis and Structure of Two New Cadmium Carbonates at Extreme Conditions. Cryst. Growth Des. 2024, 24, 4143–4150. 10.1021/acs.cgd.4c00225.

[ref19] Smith G. P.; Holroyd S. E.; Reid D. C. W.; Gordon K. C. Raman imaging processed cheese and its components. J. Raman Spectrosc. 2017, 48, 374–383. 10.1002/jrs.5054.

[ref20] Liermann H.-P.; et al. The extreme conditions beamline P02.2 and the extreme conditions science infrastructure at PETRA III. J. Synchrotron Radiat. 2015, 22, 908–924. 10.1107/S1600577515005937. 26134794 PMC4489534

[ref21] Rothkirch A.; Gatta G. D.; Meyer M.; Merkel S.; Merlini M.; Liermann H.-P. Single-crystal diffraction at the extreme conditions beamline P02.2: procedure for collecting and analyzing high-pressure single-crystal data. J. Synchrotron Radiat. 2013, 20, 711–720. 10.1107/S0909049513018621. 23955034

[ref22] Bykova E.; Aprilis G.; Bykov M.; Glazyrin K.; Wendt M.; Wenz S.; Liermann H.-P.; Roeh J. T.; Ehnes A.; Dubrovinskaia N.; Dubrovinsky L. Single-crystal diffractometer coupled with double-sided laser heating system at the extreme conditions beamline P02.2 at PETRAIII. Rev. Sci. Instrum. 2019, 90, 073907 10.1063/1.5108881. 31370445

[ref23] Prescher C.; Prakapenka V. B. DIOPTAS: a program for reduction of two-dimensional X-ray diffraction data and data exploration. High Pressure Res. 2015, 35, 223–230. 10.1080/08957959.2015.1059835.

[ref24] Toby B. H.; Von Dreele R. B. GSAS-II: the genesis of a modern open-source all purpose crystallography software package. J. Appl. Crystallogr. 2013, 46, 544–549. 10.1107/S0021889813003531.

[ref25] Aslandukov A.; Aslandukov M.; Dubrovinskaia N.; Dubrovinsky L. Domain Auto Finder (DAFi) program: the analysis of single-crystal X-ray diffraction data from polycrystalline samples. J. Appl. Crystallogr. 2022, 55, 1383–1391. 10.1107/S1600576722008081. 36249501 PMC9533752

[ref26] Dolomanov O. V.; Bourhis L. J.; Gildea R. J.; Howard J. A. K.; Puschmann H. OLEX2: a complete structure solution, refinement and analysis program. J. Appl. Crystallogr. 2009, 42, 339–341. 10.1107/S0021889808042726.

[ref27] Sheldrick G. M. SHELXT – integrated space-group and crystal-structure determination. Acta Crystallogr., Sect. A:Found. Adv. 2015, 71, 3–8. 10.1107/S2053273314026370. 25537383 PMC4283466

[ref28] Sheldrick G. M. Crystal structure refinement with SHELXL. Acta Crystallogr., Sect. C:Struct. Chem. 2015, 71, 3–8. 10.1107/S2053229614024218. 25567568 PMC4294323

[ref29] Hohenberg P.; Kohn W. Inhomogeneous electron gas. Phys. Rev. 1964, 136, B864–B871. 10.1103/PhysRev.136.B864.

[ref30] Perdew J. P.; Burke K.; Ernzerhof M. Generalized gradient approximation made simple. Phys. Rev. Lett. 1996, 77, 3865 10.1103/PhysRevLett.77.3865. 10062328

[ref31] Clark S. J.; Segall M. D.; Pickard C. J.; Hasnip P. J.; Probert M. I. J.; Refson K.; Payne M. C. First principles methods using CASTEP. Z. fur Krist. - Cryst. Mater. 2005, 220, 567–570. 10.1524/zkri.220.5.567.65075.

[ref32] Lejaeghere K.; Bihlmayer G.; Björkman T.; Blaha P.; Blügel S.; Blum V.; Caliste D.; Castelli I. E.; Clark S. J.; Dal Corso A.; et al. Reproducibility in density functional theory calculations of solids. Science 2016, 351, 1 10.1126/science.aad3000. 27013736

[ref33] Monkhorst H. J.; Pack J. D. Special points for Brillouin-zone integrations. Phys. Rev. B 1976, 13, 5188–5192. 10.1103/PhysRevB.13.5188.

[ref34] Dudarev S. L.; Botton G. A.; Savrasov S. Y.; Humphreys C. J.; Sutton A. P. Electron-energy-loss spectra and the structural stability of nickel oxide: An LSDA+U study. Phys. Rev. B 1998, 57, 1505–1509. 10.1103/PhysRevB.57.1505.

[ref35] Cococcioni M.; De Gironcoli S. Linear response approach to the calculation of the effective interaction parameters in the LDA + U method. Phys. Rev. B 2005, 71, 035105 10.1103/PhysRevB.71.035105.

[ref36] Baroni S.; de Gironcoli S.; Dal Corso A.; Giannozzi P. Phonons and related crystal properties from density-functional perturbation theory. Rev. Mod. Phys. 2001, 73, 515 10.1103/RevModPhys.73.515.

[ref37] Refson K.; Tulip P. R.; Clark S. J. Variational density-functional perturbation theory for dielectrics and lattice dynamics. Phys. Rev. B 2006, 73, 155114 10.1103/PhysRevB.73.155114.

[ref38] Miwa K. Prediction of Raman spectra with ultrasoft pseudopotentials. Phys. Rev. B 2011, 84, 094304 10.1103/PhysRevB.84.094304.

[ref39] Santoro M.; Lin J.-f.; Mao H.-k.; Hemley R. J. In situ high P-T Raman spectroscopy and laser heating of carbon dioxide. J. Chem. Phys. 2004, 121, 2780–2787. 10.1063/1.1758936. 15281882

[ref40] Iota V.; Yoo C. S.; Cynn H. Quartzlike Carbon Dioxide: An Optically Nonlinear Extended Solid at High Pressures and Temperatures. Science 1999, 283, 1510–1513. 10.1126/science.283.5407.1510. 10066168

[ref41] Scelta D.; Dziubek K. F.; Ende M.; Miletich R.; Mezouar M.; Garbarino G.; Bini R. Extending the stability field of polymeric carbon dioxide phase V beyond the Earth’s geotherm. Phys. Rev. Lett. 2021, 126, 065701 10.1103/PhysRevLett.126.065701. 33635684

[ref42] Kreisel K. A.; Yap G. P. A.; Dmitrenko O.; Landis C. R.; Theopold K. H. The Shortest Metal-Metal Bond Yet: Molecular and Electronic Structure of a Dinuclear Chromium Diazadiene Complex. J. Am. Chem. Soc. 2007, 129, 14162–14163. 10.1021/ja076356t. 17967028

[ref43] Shannon R. D. Revised effective ionic radii and systematic studies of interatomic distances in halides and chalcogenides. Acta Crystallogr., Sect. A 1976, 32, 751–767. 10.1107/S0567739476001551.

[ref44] Momma K.; Izumi F. VESTA: a three-dimensional visualization system for electronic and structural analysis. J. Appl. Crystallogr. 2008, 41, 653–658. 10.1107/S0021889808012016.

[ref45] Gonzalez-Platas J.; Alvaro M.; Nestola F.; Angel R. EosFit7-GUI: a new graphical user interface for equation of state calculations, analyses and teaching. J. Appl. Crystallogr. 2016, 49, 1377–1382. 10.1107/S1600576716008050.

[ref46] Shim S.-H.; Duffy T. S.; Jeanloz R.; Yoo C.-S.; Iota V. Raman spectroscopy and x-ray diffraction of phase transitions in Cr_2_O_3_ to 61 GPa. Phys. Rev. B 2004, 69, 144107 10.1103/PhysRevB.69.144107.

[ref47] Kantor A.; Kantor I.; Merlini M.; Glazyrin K.; Prescher C.; Hanfland M.; Dubrovinsky L. High-pressure structural studies of eskolaite by means of single-crystal X-ray diffraction. Am. Mineral. 2012, 97, 1764–1770. 10.2138/am.2012.4103.

[ref48] Golosova N.; Kozlenko D.; Kichanov S.; Lukin E.; Liermann H.-P.; Glazyrin K.; Savenko B. Structural and magnetic properties of Cr_2_O_3_ at high pressure. J. Alloys Compd. 2017, 722, 593–598. 10.1016/j.jallcom.2017.06.140.

[ref49] Dobin A. Y.; Duan W.; Wentzcovitch R. M. Magnetostructural effects and phase transition in Cr_2_O_3_ under pressure. Phys. Rev. B 2000, 62, 11997–12000. 10.1103/PhysRevB.62.11997.

[ref50] Wessel C.; Dronskowski R. A first-principles study on chromium sesquioxide, Cr_2_O_3_. J. Solid State Chem. 2013, 199, 149–153. 10.1016/j.jssc.2012.12.019.

[ref51] Mashimo T.; Tsumoto K.; Nakamura K.; Noguchi Y.; Fukuoka K.; Syono Y. High-pressure phase transformation of corundum (α-Al_2_O_3_) observed under shock compression. Geophys. Res. Lett. 2000, 27, 2021–2024. 10.1029/2000GL008490.

[ref52] Funamori N.; Jeanloz R. High-Pressure Transformation of Al_2_O_3_. Science 1997, 278, 1109–1111. 10.1126/science.278.5340.1109.

[ref53] Lin J.-F.; Degtyareva O.; Prewitt C. T.; Dera P.; Sata N.; Gregoryanz E.; Mao H.-k.; Hemley R. J. Crystal structure of a high-pressure/high-temperature phase of alumina by in situ X-ray diffraction. Nat. Mater. 2004, 3, 389–393. 10.1038/nmat1121. 15146173

[ref54] Bykova E.; Dubrovinsky L.; Dubrovinskaia N.; Bykov M.; McCammon C.; Ovsyannikov S. V.; Liermann H. P.; Kupenko I.; Chumakov A. I.; Rüffer R.; Hanfland M.; Prakapenka V. Structural complexity of simple Fe_2_O_3_ at high pressures and temperatures. Nat. Commun. 2016, 7, 10661 10.1038/ncomms10661. 26864300 PMC4753252

