# Supplementary material for: Cr3+-Containing Carbonates and Cr2O3-Pbcn at Extreme Conditions
Source: Inorg Chem. 2025 Mar 4;64(10):4996–5003. doi: 10.1021/acs.inorgchem.4c05003 (PMC11920955; doi:10.1021/acs.inorgchem.4c05003)
Supplement: Supplementary file 1 — ic4c05003_si_001.pdf [file ic4c05003_si_001.pdf]

Supplementary Material: Cr^{3+}-containing carbonates and Cr_2O_3-Pbcn at extreme conditions

Yu. Wang,[*,†] Lkhamsuren Bayarjargal,[†] Maxim Bykov,[‡] Elena Bykova,[†] Dominik Spahr,[†] Konstantin Glazyrin,[¶] Victor Milman,[§] and Bjoern Winkler[†]

†*Institute of Geosciences, Goethe University Frankfurt, 60438 Frankfurt, Germany*

‡*Institute of Inorganic and Analytical Chemistry, Goethe University Frankfurt, 60438 Frankfurt, Germany*

¶*Deutsches Elektronen-Synchrotron DESY, Notkestr. 85, 22607 Hamburg, Germany*

§*Dassault Systemes BIOVIA, 22 Cambridge Science Park, Cambridge CB4 0FJ, United Kingdom*

E-mail: yu.wang@kristall.uni-frankfurt.de

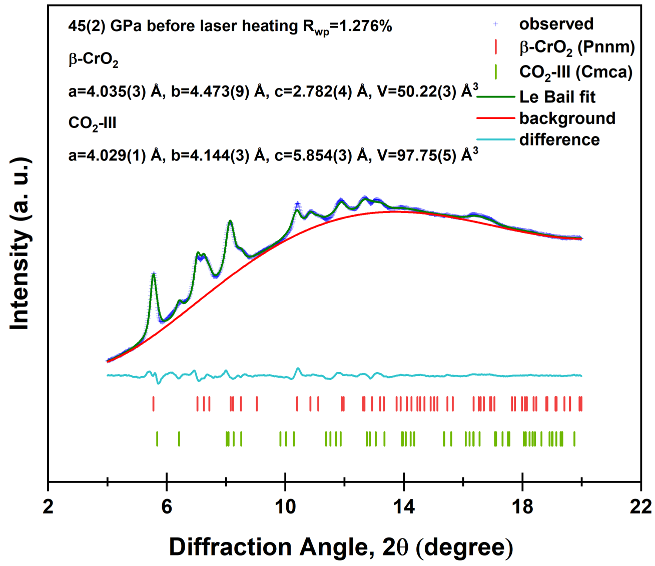

Figure S1: Blue crosses represent the experimental observed data. Green solid line represents a Le Bail fit. Red solid line shows the fitted background. The cyan solid line shows the difference between Le Bail fit and observed data. Red ticks represent the positions calculated by CrO_2-$Pnnm$. Green ticks represent the positions calculated by CO_2-$Cmca$ (CO_2-III). The R-value of total weighted pattern is $R_{wp} = 1.27\%$ according to the Le Bail fit.

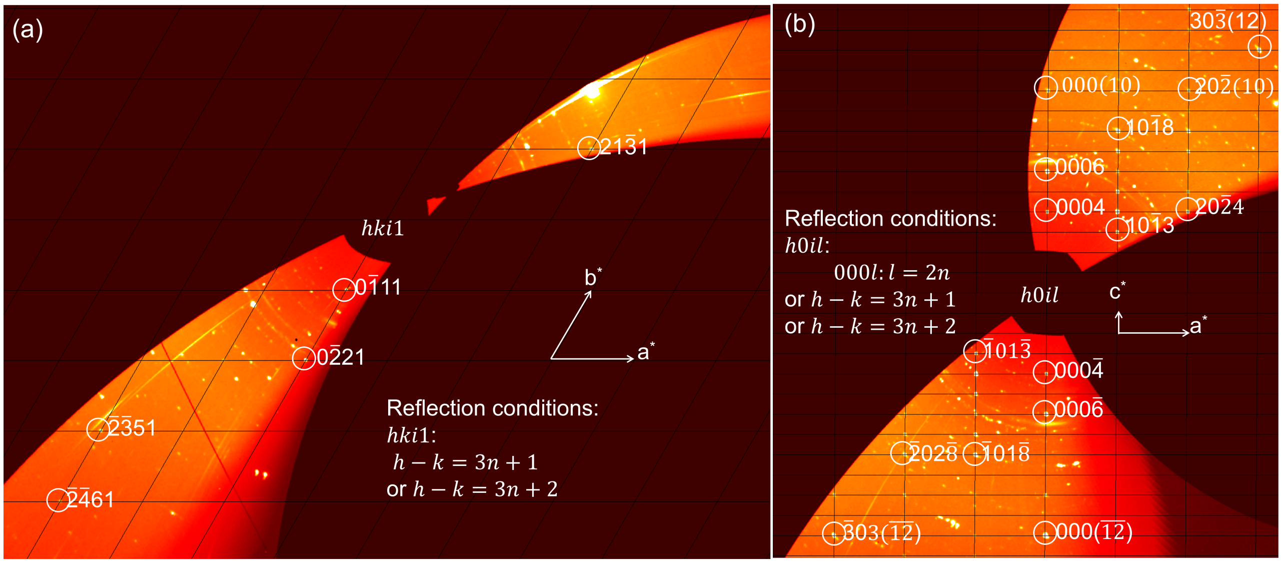

Figure S2: The slices of reciprocal space visualized by CrysAlisPro software package with attributed diffraction spots, marked by white circles. The arrows show the reciprocal vectors. The black solid lines represent the reciprocal lattice grid. (a) ($hki1$) slice. (b) ($h0il$) slice.

To confirm the space group of $Cr_2[CO_3]_3$ structure model, we unwarped the reciprocal

lattice generated from the single crystal dataset and used it for validating the reflection conditions for this space group (Figure S2). The general condition for Miller indices $000l$ is $l = 2n$, while for $hkil$ in which $l \neq 2n$, the reflection conditions should be satisfied $h - k = 3n + 1$ or $3n + 2$ (as shown in Figure S2). All the reflections shown in the Figure S2 are consisted with the rules, indicating the chosen of $P6_3/m$ space group is correct.

Table S1: Summary on $Cr_2[CO_3]_3$-$P6_3/m$ experimental data, structure solution and refinement details, together with DFT calculations at 45(2) GPa.

Crystal data	EXP.	DFT
Chemical formula	$Cr_2[CO_3]_3$	
M_R (g/mol)	284.03	
Crystal system, space group	hexagonal, $P6_3/m$	
Pressure (GPa)	45(2)	45
Temperature (K)	293	0
a (Å)	4.375(4)	4.3484
c (Å)	13.022(2)	12.815
V (Å^3)	215.89(18)	209.849
Z	2	
Data collection		
Radiation	$\lambda = 0.2900$ Å	
T_{min}, T_{max}	0.705, 1.000	
No. of measured reflections	635	
No. of independent reflections	221	
Reflections $I > 2\sigma I$	131	
R_{int}	0.052	
$(\sin\Theta/\lambda_{max})$(Å^{-1})	1.010	
Refinement		
$R_1[F^2 > 2\sigma(F^2)], wR_2(F^2)$	0.064, 0.178	
No. of independent reflections	221	
No. of parameters	26	
$\Delta\rho_{\max}$,$\Delta\rho_{\min}$ (e Å^{-3})	1.50, -1.02	

Table S2: Fractional atomic coordinates and isotropic displacement parameters of $Cr_2[CO_3]_3$-$P6_3/m$ at about 45 GPa.

	**x**	**y**	**z**	$U^*_{\mathbf{iso}}/U_{\mathbf{eq}}$	**Occ.**	**Site**
Cr1	1.000000	1.000000	0.50000	0.0111 (6)	1	2b
Cr2	0.666667	0.333333	0.32319 (17)	0.0089 (7)	0.5	4f
C1	0.333333	0.666667	0.4149 (6)	0.013 (2)	1	4f
C2	0.000000	0.000000	0.250000	0.008 (3)	1	2a
O1	0.6210 (13)	0.6618 (11)	0.4160 (2)	0.0107 (9)	1	12i
O2	0.2836 (19)	0.291 (2)	0.250000	0.0094 (13)	1	6h

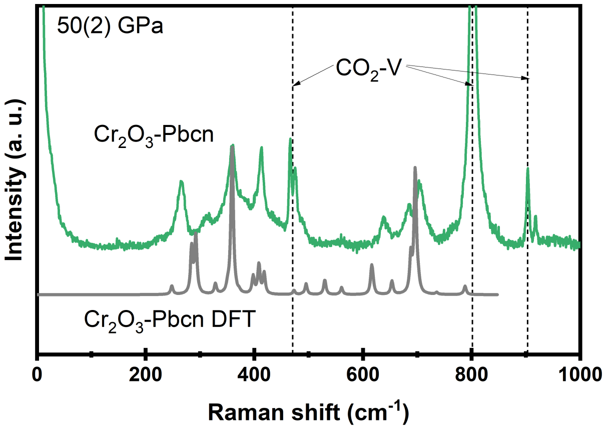

Figure S3: Raman spectra of Cr_2O_3 compared in experiment and DFT at 50(2) GPa.

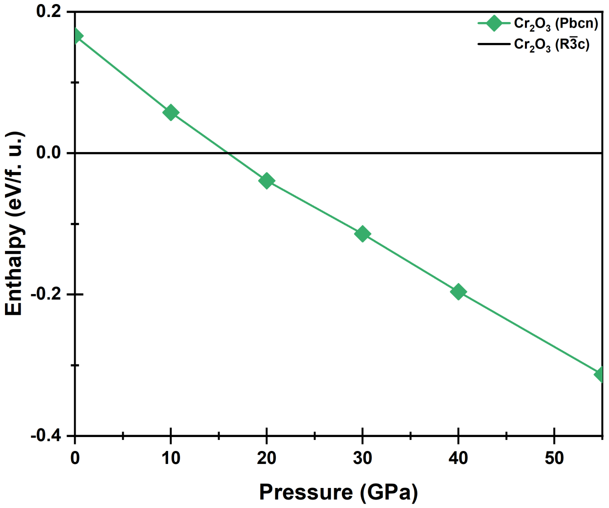

Figure S4: Pressure dependence of the differences between the enthalpies of Cr_2O_3 in *Pbcn* and $R\bar{3}c$ space groups.

Table S3: Summary on $Cr_2[C_2O_5][CO_3]_2$-$C2/c$ experimental data, structure solution and refinement details, together with DFT calculations at 45(2) GPa.

Crystal data	EXP.	DFT
Chemical formula	Cr2[C2O5][CO3]2	
M_R (g/mol)	328.04	
Crystal system, space group	Monoclinic, $C2/c$	
Pressure (GPa)	45(2)	45
Temperature (K)	293	0
a (Å)	11.3814(19)	11.3542
b (Å)	4.297(2)	4.3566
c (Å)	10.8709(13)	10.9163
$\beta(°)$	106.994(13)	107.386
V (Å^3)	508.4(3)	515.31
Z	4	
Data collection		
Radiation	$\lambda = 0.2912$ Å	
T_{min}, T_{max}	0.957, 1.000	
No. of measured reflections	877	
No. of independent reflections	597	
Reflections $I>2\sigma I$	354	
R_{int}	0.032	
$(\sin\Theta/\lambda_{max})$(Å^{-1})	1.044	
Refinement		
$R_1[F^2 > 2\sigma(F^2)], wR_2(F^2)$	0.068, 0.173	
No. of independent reflections	597	
No. of parameters	73	
$\Delta\rho_{\max}$,$\Delta\rho_{\min}$ (e Å^{-3})	0.95, -0.80	

Table S4: $Cr_2[CO_3][O]_2$-*Pbcn* crystal data compared with DFT calculations and structure refinement details at 50(2) GPa.

Crystal data	EXP.	DFT
Chemical formula	$Cr_2[CO_3][O]_2$	
M_R (g/mol)	196.01	
Crystal system, space group	Orthorhombic, *Pbcn*	
Pressure (GPa)	50(2)	55
Temperature (K)	293	0
a (Å)	11.215(4)	11.1712
b (Å)	4.4193(5)	4.4096
c (Å)	4.9472(10)	4.8921
V (Å^3)	254.20(10)	240.98
Z	4	
Data collection		
Radiation	$\lambda = 0.2912$ Å	
T_{min}, T_{max}	0.574, 1.000	
No. of measured reflections	1170	
No. of independent reflections	561	
Reflections $I > 2\sigma I$	371	
R_{int}	0.028	
$(\sin\Theta/\lambda_{max})$(Å^{-1})	1.048	
Refinement		
$R_1[F^2 > 2\sigma(F^2)], wR_2(F^2)$	0.047, 0.132	
No. of independent reflections	561	
No. of parameters	38	
$\Delta\rho_{\max}$,$\Delta\rho_{\min}$ (e Å^{-3})	1.27, -1.23	

Table S5: Cr_2O_3-*Pbcn* crystal data compared with DFT calculations and structure refinement details at 50(2) GPa.

Crystal data	EXP.	DFT
Chemical formula	Cr_2O_3	
M_R (g/mol)	152.00	
Crystal system, space group	Orthorhombic, *Pbcn*	
Pressure (GPa)	50(2)	50
Temperature (K)	293	0
a (Å)	6.823(4)	6.8433
b (Å)	4.784(5)	4.8263
c (Å)	5.010(2)	5.0187
V (Å^3)	163.54 (10)	165.75672
Z	4	
Data collection		
Radiation	$\lambda = 0.2912$ Å	
T_{min}, T_{max}	0.438, 1.000	
No. of measured reflections	762	
No. of independent reflections	306	
Reflections $I > 2\sigma I$	240	
R_{int}	0.013	
$(\sin\Theta/\lambda_{max})$(Å^{-1})	1.057	
Refinement		
$R_1[F^2 > 2\sigma(F^2)], wR_2(F^2)$	0.026, 0.077	
No. of independent reflections	306	
No. of parameters	24	
$\Delta\rho_{\max}$,$\Delta\rho_{\min}$ (e Å^{-3})	0.94, -0.85	

Table S6: Summary on geometric parameters of octahedron in $Cr_2[C_2O_5][CO_3]_2$ and $Al_2[C_2O_5][CO_3]_2$ at about 45 GPa.

	$Cr_2[C_2O_5][CO_3]_2$	$Al_2[C_2O_5][CO_3]_2$
Polyhedron volume (Å^3)	9.0	7.9
Average bond length (Å)	1.89	1.81
Quadratic elongation[1]	1.002	1.0018
Bond angle variance[2] (deg.^{2})	6.983	4.338

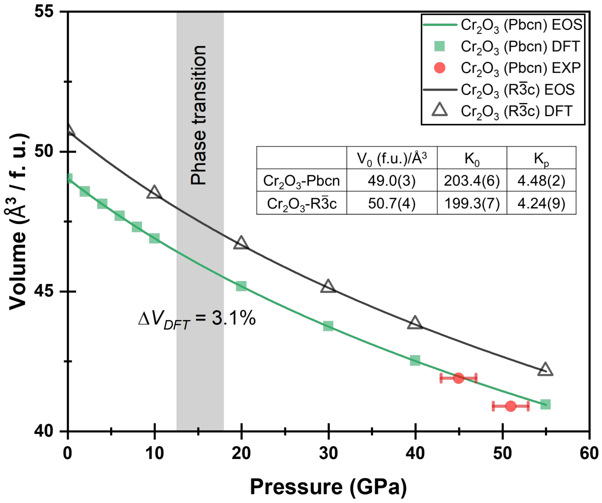

Figure S5: Equation of the state of Cr_2O_3 at high pressures by DFT,[3] shown together with experimental data.

Table S7: Summary of high-pressure reactions and products.

Starting materials	Pressure	Heating conditions	Products
$CrO_2 + CO_2$	45 GPa	~1500 K, 20 s	$Cr_2[CO_3]_3$-$P6_3/m$
	45 GPa	~1500 K, 10 min	$Cr_2[C_2O_5][CO_3]_3$-$C2/c$
$Cr_2O_3 + CO_2$	55 GPa	1500-2000 K, 20 s	$Cr_2[CO_3]_3$-$P6_3/m$
	55 GPa	1500-2000 K, 20 s	$Cr_2[CO_3][O]_2$-$Pbcn$
	55 GPa	1500-2000 K, 20 s	Cr_2O_3-$Pbcn$
	45 GPa	1500-2000 K, 20 s	$Cr_2[CO_3]_3$-$P6_3/m$
	45 GPa	1500-2000 K, 10 min	$Cr_2[C_2O_5][CO_3]_3$-$C2/c$
	55 GPa	1500-2000 K, 20 s	Cr_2O_3-$Pbcn$

Table S8: Cr_2O_3-*Pbcn* geometric parameters from experiments and DFT calculations at 50(2) GPa.

Coordination		EXP.	DFT
Cr1	x	0.39286	0.39017
	y	0.25121	0.25080
	z	0.04103	0.03437
O1	x	0.00000	0.00000
	y	0.06020	0.05283
	z	0.25000	0.25000
O2	x	0.34500	0.34722
	y	0.10660	0.10548
	z	0.39530	0.39044
Distance (Å)			
Cr-O	Cr1-O1	1.9535(13)	1.965
	Cr1-$O1^{iii}$	1.8640(9)	1.871
	Cr1-O2	1.8895(12)	1.888
	Cr1-$O2^{ii}$	1.944(2)	1.966
	Cr1-$O2^{iv}$	1.905(2)	1.908
	Cr1-$O2^{v}$	1.9327(12)	1.942

Table S9: High pressure behavior of Cr_2O_3 in previous study and this work.

	Laser Heating	Temperature [K]	Phase
Cr_2O_3 [4]	15 GPa, no LH	–	*I2/a*
Cr_2O_3 [4]	30 GPa	1500-2000	*Pbcn/Pbnm*
Cr_2O_3 [5]	70 GPa, no LH	–	$R\bar{3}c$
Cr_2O_3 this work	45 GPa<br>55 GPa	1500-2000	*Pbcn*
Cr^{3+}-Al_2O_3 [6,7]	~100 GPa	~1000	*Pbcn*
Al_2O_3 [8]	79 GPa	1173	*Pbcn*
Al_2O_3 [9]	150 GPa	2000	*Pbnm*
α-Fe_2O_3 [10]	40 GPa	~2000	*Pbcn*

References

(1) Goto, T.; Ahrens, T. J.; Rossman, G. R. Absorption spectra of Cr^{3+} in Al_2O_3 under shock compression. *Physics and Chemistry of Minerals* **1979**, *4*, 253–263.

(2) Hazen, R. M.; Downs, R. T.; Prewitt, C. T. Principles of Comparative Crystal Chemistry. *Reviews in Mineralogy and Geochemistry* **2000**, *41*, 1–33.

(3) Gonzalez-Platas, J.; Alvaro, M.; Nestola, F.; Angel, R. *EosFit7-GUI* : a new graphical user interface for equation of state calculations, analyses and teaching. *Journal of Applied Crystallography* **2016**, *49*, 1377–1382.

(4) Shim, S.-H.; Duffy, T. S.; Jeanloz, R.; Yoo, C.-S.; Iota, V. Raman spectroscopy and x-ray diffraction of phase transitions in Cr_2O_3 to 61 GPa. *Physical Review B* **2004**, *69*, 144107.

(5) Kantor, A.; Kantor, I.; Merlini, M.; Glazyrin, K.; Prescher, C.; Hanfland, M.; Dubrovinsky, L. High-pressure structural studies of eskolaite by means of single-crystal X-ray diffraction. *American Mineralogist* **2012**, *97*, 1764–1770.

(6) Funamori, N.; Jeanloz, R. High-Pressure Transformation of Al_2O_3. *Science* **1997**, *278*, 1109–1111.

(7) Lin, J.-F.; Degtyareva, O.; Prewitt, C. T.; Dera, P.; Sata, N.; Gregoryanz, E.; Mao, H.-k.; Hemley, R. J. Crystal structure of a high-pressure/high-temperature phase of alumina by in situ X-ray diffraction. *Nature Materials* **2004**, *3*, 389–393.

(8) Mashimo, T.; Tsumoto, K.; Nakamura, K.; Noguchi, Y.; Fukuoka, K.; Syono, Y. High-pressure phase transformation of corundum (α-Al_2O_3) observed under shock compression. *Geophysical Research Letters* **2000**, *27*, 2021–2024.

(9) Ono, S.; Oganov, A. R.; Koyama, T.; Shimizu, H. Stability and compressibility of the high-pressure phases of Al_2O_3 up to 200 GPa: Implications for the electrical conduc-

tivity of the base of the lower mantle. *Earth and Planetary Science Letters* **2006**, *246*, 326–335.

(10) Bykova, E.; Dubrovinsky, L.; Dubrovinskaia, N.; Bykov, M.; McCammon, C.; Ovsyannikov, S. V.; Liermann, H. P.; Kupenko, I.; Chumakov, A. I.; Rüffer, R.; Hanfland, M.; Prakapenka, V. Structural complexity of simple Fe_2O_3 at high pressures and temperatures. *Nature Communications* **2016**, *7*, 10661.
